# Tumor boundary delineation in IDH-wildtype glioblastoma: A prospective comparative analysis of ^11^C-MET PET and FLAIR MRI in surgical decision-making

**DOI:** 10.1093/nop/npag023

**Published:** 2026-03-24

**Authors:** Maria Pia Tropeano, Zefferino Rossini, Andrea Pizzi, Ettore Bresciani, Andrea Franzini, Beatrice Claudia Bono, Pierina Navarria, Elena Clerici, Matteo Simonelli, Marta Scorsetti, Letterio Salvatore Politi, Federico Pessina

**Affiliations:** Neurosurgery Department, IRCCS Humanitas Research Hospital, Milan, Italy; Neurosurgery Department, IRCCS Humanitas Research Hospital, Milan, Italy; Neurosurgery Department, IRCCS Humanitas Research Hospital, Milan, Italy; Department of Biomedical Sciences, Humanitas University, Milan, Italy; Neurosurgery Department, IRCCS Humanitas Research Hospital, Milan, Italy; Department of Biomedical Sciences, Humanitas University, Milan, Italy; Neurosurgery Department, IRCCS Humanitas Research Hospital, Milan, Italy; Neurosurgery Department, IRCCS Humanitas Research Hospital, Milan, Italy; Radiotherapy and Radiosurgery Department, IRCCS Humanitas Research Hospital, Milan, Italy; Radiotherapy and Radiosurgery Department, IRCCS Humanitas Research Hospital, Milan, Italy; Department of Biomedical Sciences, Humanitas University, Milan, Italy; Radiotherapy and Radiosurgery Department, IRCCS Humanitas Research Hospital, Milan, Italy; Department of Medical Oncology and Hematology, Humanitas Clinical and Research Center—IRCCS, Humanitas Cancer Center, Milan, Italy; Department of Biomedical Sciences, Humanitas University, Milan, Italy; Department of Neuroradiology, IRCCS Humanitas Research Hospital, Milan, Italy; Neurosurgery Department, IRCCS Humanitas Research Hospital, Milan, Italy; Department of Biomedical Sciences, Humanitas University, Milan, Italy

**Keywords:** ^11^C-MET PET, glioblastoma, high grade gliomas, MRI, PET, supramaximal resection

## Abstract

**Background:**

Supramaximal resection (SUPR) in IDH-wildtype glioblastoma (GBM) aims to improve survival by extending resection beyond contrast-enhancing lesions into non-enhancing FLAIR-hyperintense regions. The role of metabolically guided resection with ^11^C-methionine positron emission tomography (^11^C-MET PET) as an alternative approach has not been systematically investigated. This study compares volumetric and clinical outcomes of ^11^C-MET PET versus MRI guidance in IDH-wildtype GBM.

**Methods:**

We performed a prospective, single-center study (2020-2024) including patients undergoing PET-guided resection for newly diagnosed, contrast-enhancing GBM. All patients received preoperative MRI and ^11^C-MET PET, followed by early postoperative imaging. Survival outcomes were compared with those of a matched SUPR, NTR (Near-total resection), and CR (Complete resection) cohort, according to the updated RANO criteria.

**Results:**

In all cases, preoperative PET volume exceeded the contrast-enhancing (CE) volume. Spatial overlap between PET-based volume and FLAIR tumor volume (FLAIR-TV) was observed in 91.2% of cases, while in 8.8% the PET volume was larger. Strong correlations were found between FLAIR-TV and PET volumes (*r*  =  0.90) and between CE and PET volumes (*r*  =  0.84). No baseline differences were identified between groups in sex, age, MGMT promoter methylation, tumor location, or preoperative Karnofsky Performance Status. Median progression-free survival (PFS) and overall survival (OS) were significantly longer in the SUPR group compared with the PET group (PFS: 20 vs. 14.9 months, *P*  =  .0003; OS: 24 vs. 21.9 months, *P*  =  .025), with the NTR group (PFS: 20 vs 11 months, *P*  <  .001; OS: 24 vs 15 months, *P*  =  .001) and CR group (PFS: 20 vs 13 months, *P*  <  .001; OS 24 vs 17 months, *P*  =  .001).

**Conclusions:**

^11^C-MET PET effectively delineates infiltrative margins and detects aggressive subclones. However, SUPR provides superior survival compared with PET-guided resections alone, supporting PET as a complementary tool rather than a stand-alone modality in surgical planning for IDH-wildtype GBM.

Key Points

- Maximizing the extent of resection (EOR) is crucial for improving outcomes in IDH-wildtype glioblastoma due to its highly infiltrative nature.
- Supramaximal resection (SUPR), which includes non-enhancing FLAIR-hyperintense areas, is encouraged by updated RANO guidelines to better address infiltrative margins.
- ^11^C-MET PET offers metabolic insights beyond standard MRI, potentially improving tumor margin delineation by identifying active tumor regions not visible on conventional imaging.
- This study prospectively compares ^11^C-MET PET and MRI in surgical planning to evaluate which method more effectively improves patient outcomes.

Importance of the Study

This study provides a direct, prospective comparison between metabolic and anatomical imaging for surgical planning in the treatment of glioblastoma. By analyzing the impact of PET-guided versus FLAIR-guided resections on patient survival, the research addresses a critical gap in neuro-oncology. The findings support the integration of metabolic imaging into surgical planning while highlighting the limitations of relying on PET alone. This could inform future guidelines, optimize surgical strategies, and ultimately improve outcomes for patients with aggressive brain tumors.

Maximizing the extent of resection (EOR) remains one of the most effective therapeutic strategies in IDH-wildtype glioblastoma (GBM), which are known for their highly infiltrative behavior and dismal prognosis.^1^ Recent guidelines from the Response Assessment in Neuro-Oncology (RANO) group have introduced the concept of supramaximal resection (SUPR), which encourages the removal of not only contrast-enhancing tissue but also adjacent non-enhancing tumor regions, typically identified on FLAIR imaging, while preserving functional integrity.^2^^,^^3^ This approach aims to address the infiltrative margins of GBM that often harbor treatment-resistant subclonal populations.

Concurrently, metabolic imaging with radiolabeled amino acids, such as ^11^C-methionine positron emission tomography (^11^C-MET PET), has emerged as a complementary tool to conventional MRI.^4–7^ By targeting amino acid transporters upregulated in neoplastic cells, MET-PET can highlight areas of metabolic activity beyond the contrast-enhancing core, potentially offering a more refined map of tumor biology.^8–10^ However, it remains unclear whether this functional information provides superior surgical guidance compared to anatomical imaging alone, particularly in the context of aggressive surgical strategies like SUPR.

To date, no studies have directly compared the volumetric and clinical implications of using ^11^C-MET PET versus FLAIR MRI to define the surgical roadmap in GBM. Furthermore, whether PET-guided resections offer a survival benefit over resections extending into FLAIR hyperintense zones remains a matter of ongoing debate.

In this prospective study, we investigated the role of ^11^C-MET PET in comparison with MRI for preoperative planning in IDH-wildtype GBM. We further examined the clinical outcomes of patients undergoing PET-guided resection versus MRI-guided resection, as defined by the updated RANO criteria.^2^ We aimed to determine whether metabolic imaging provides a clear advantage in delineating surgical targets or whether anatomical criteria alone remain more effective in improving survival.

## Methods

### Study Design and Patient Population

This was a single-center, observational, prospective study.

We included patients with newly diagnosed, histologically confirmed glial tumors with contrast-enhancing lesions who underwent surgical resection at our Institution between January 2020 and January 2024. All patients had pre-operative imaging with ^11^C-MET PET and MRI. Early postoperative MRI and ^11^C-MET PET assessed the extent of resection.

Exclusion criteria included: (1) age  <  18 years; (2) previous surgical resection or biopsy; (3) IDH mutation; (4) missing pre- or post-operative MRI or PET data; (5) previous radiotherapy and/or chemotherapy; (6) incomplete PET-guided resection.

We collected demographic, clinical, histomolecular, imaging, treatment, and outcome data for each included patient. This study was approved by the Humanitas Research Hospital ethics committee [approval n. 19/21] and was conducted in accordance with the Declaration of Helsinki.

### Radiological Data

#### Magnetic resonance imaging

For all patients, volumetric MRI sequences were acquired before surgery using a 3 Tesla Siemens MAGNETOM Verio MRI scanner (Siemens Medical Systems). The preoperative MRI protocol included T1-weighted pre- and post-gadolinium, T2-weighted, and FLAIR scans and, in most cases, DWI, fMRI, and DTI were also performed. Postoperative MRI at 48 h and at 2 months were obtained to estimate the extent of resection. All patients in the MRI group were therefore classified into extent of resection categories by the 2022 RANO-Resect classification: Near-Total Resection (NTR) was thus defined as residual contrast-enhancing volume >1 cm^3^ regardless of FLAIR disease at postoperative images, while Complete Resection (CR) encompassed patients without any residual CE enhancement and postoperative FLAIR volume >5 cm^3^; lastly, Supramaximal Resection (SUPR) included all patients with residual FLAIR-disease <5 cm^3^ without evidence of contrast-enhancing tumor tissue. All patients with EOR below such definitions were excluded from analysis.

#### ^11^C-MET PET imaging

All patients underwent preoperative ^11^C-MET PET imaging within 15 days before surgery. L-[^11^C-methyl]-methionine was synthesized on-site. Three-dimensional PET/CT images were acquired according to the hospital protocol with either a GE Discovery 690 (GE Healthcare) or a Siemens Biograph 6 (Siemens Medical Systems) scanner.

### Histopathological Analysis

All cases were histologically and molecularly evaluated and classified according to the latest CNS WHO 2021 guidelines.^11^^,^^12^ The IDH status was evaluated by immunohistochemical staining in all patients, with an additional molecular assay for negative cases.

MGMT promoter methylation status was assessed through DNA extraction from tumor tissue and pyrosequencing analysis (Diatech Pharmacogenetics, MGMTplus, CE, and IVD validated). At our institution, values above 5% are classified as positive for MGMT promoter methylation, while values equal to or under such threshold are considered negative.

### Postoperative Treatments

To define the appropriate therapy, each patient was evaluated by a multidisciplinary team including neurosurgeons, neuro-oncologists, neuroradiologists, and radiotherapists. Surgery was followed, within 4 to 6 weeks, by radiotherapy (RT) with concomitant temozolomide (TMZ) and maintenance TMZ alone, as per the Stupp regimen.^1^^,^^13^

### Statistical Analysis

Standard descriptive analyses were used to describe patients’ characteristics; continuous variables were computed as means with standard deviation or median values with ranges according to the normality of distribution as assessed with the Shapiro–Wilk test; categorical variables were computed as numbers and frequencies. For the outcome analysis, Kaplan-Meyer curves were calculated considering the date of surgery and the date of disease progression for the PFS, and the patient’s exitus for the OS. Proportional Hazard Cox regression univariable analysis was conducted to test the association of a variable set of factors known to be associated with glioblastoma outcome, including gender, age, MGMT promoter methylation status, location, and postoperative KPS. All independent variables with a *P* value under .2 were then submitted to a multivariable analysis, both for PFS and OS. Results were then expressed as Hazard Ratio (HR), with 95% confidence interval (95% CI). The significance threshold was set to 0.05. All analyses were made with Stata version 18 (StataCorp, 2023. Stata Statistical Software: Release 18. College Station, TX: StataCorp LLC).

## Results

### Patient Characteristics

From January 1, 2020 to January 31, 2024, 320 adult patients with newly diagnosed gliomas were treated at our institution; 25 were excluded due to IDH mutation at immunohistochemical or molecular analysis. Patients lost at follow-up or whose preoperative and postoperative MR and PET images were not available were further excluded. Within the included sample, all patients underwent intensity-modulated radiotherapy and concomitant temozolomide as per the Stupp Protocol; subsequently, all patients underwent adjuvant temozolomide. Following the computation of volumetric analyses of all images, 117 patients were included in the MRI-guided group, of which 45 underwent Near Total Resection (NTR) and 31 Complete Resection (CR), while 41 cases were included in the Supramaximal Resection group (SUPR). A total of 34 patients with a newly diagnosed, histologically confirmed GBM IDH1 wild-type fulfilling selection criteria were included in the PET-guided group. The patient characteristics are summarized in Table 1. The mean age in the PET group was 59.4 years (range 21 to 79 years), and there were 22 (64.71%) males and 12 (35.29%) females. No significant differences were found in terms of age or sex. Methylation of MGMT promoter was observed in 22 patients (64.71%). The mean preoperative Karnofsky Performance Status (KPS) was 93.2  ±  10.1 and the mean postoperative KPS was 92.64  ±  9.60.

**Table 1.** Comparison between SUPR and PET groups

	PET group	MRI group			
		SUPR	NTR	CR	*P*
*N*	34	41	45	31	
Sex (M)	22 (64.71%)	24 (58.54%)	34 (75.56%)	23 (74.19%)	.316
Age	59.4 ± 12.3	58.3 ± 12.4	60.2 ± 11.8	63.2 ± 9.0	.493
MGMT (methylated)	22 (64.71%)	28 (68.29%)	31 (68.89%)	18 (58.06%)	.772
Location					
Near eloquent	10 (29.41%)	12 (29.27%)	33 (73.33%)	10 (32.26%)	
Eloquent	8 (23.52%)	6 (14.63%)	1 (2.22%)	7 (22.58%)	
Non eloquent	16 (47.05%)	15 (36.59%)	11 (24.44%)	14 (45.16%)	
Side (R)	15 (44.12%)	23 (56.10%)	16 (35.56%)	15 (48.39%)	.378
KPS pre	93.2 ± 10.1	90.7 ± 8.8	91.0 ± 6.4	92.6 ± 7.1	.348
Volumi pre					
CE	31.4 ± 27.9	32.4 ± 39.2	39.9 ± 37.0	30.7 ± 27.8	
FLAIR	79.9 ± 51.3 (8.13-201.34)	36.8 ± 61.1 (1.26-337.65)	32.1 ± 42.6 13.7 (1.35-159.28)	38.3 ± 22.3 35.55 (10.76-93.01)	
OS (months) (Median Q1-Q3)	21.88 (5-27)	24 (17-37)	15 (11-24)	17 (11-26)	.001
PFS (months) (Median Q1-Q3)	14.94 (12-34)	20 (12-26)	11 (7-15)	13 (8-16)	<.001

Within the entire MRI-cohort, there were 81 males (68.6%), median age was 63 years (range 21-80); the mean preoperative KPS was 91.61  ±  7.29, and the mean postoperative KPS was 89.05  ±  9.60. MGMT promoter was observed in 77 (65.8%) patients, evenly distributed between EOR classes (*P  *=  .569).

Furthermore, there was no significant difference in the distribution of lesions in eloquent regions between the PET and MRI group (*P*  =  .11).

### Volumetric Measurements

The interobserver agreement between the first 2 readers was strong (к: 0.826).

Overall, qualitative analysis of ^11^C-MET PET scans revealed all positive studies.

Semi-quantitative ^11^C-MET PET image analysis was performed in all cases. The median values of TBRmax and TBRmean were 3.24 and 1.81, respectively.

The mean preoperative CE volume was 31.4  ±  27.9 cc (range 0.42-115.13), the median preoperative ^11^C-MET PET volume was 53.55  ±  35.8 cc (range 4.547-176.47), and the median preoperative FLAIR-TV was 79.9  ±  51.3 cc (range 8.13-201.34). In all cases, preoperative ^11^C-MET PET volume>preoperative CE volume. There was a spatial overlap between the MET PET-based and FLAIR-TV in 31 (91.17%) cases. In the remaining 3 cases (8.82%), preoperative ^11^C-MET PET volume>preoperative FLAIR-TV.

Patients with methylated MGMT had a slightly higher preoperative PET volume (57.4  ±  44.3 cc) compared to non-methylated cases (46.5  ±  27.5 cc), but the difference was not statistically significant (*P*  =  .709).

**Figure 1.**
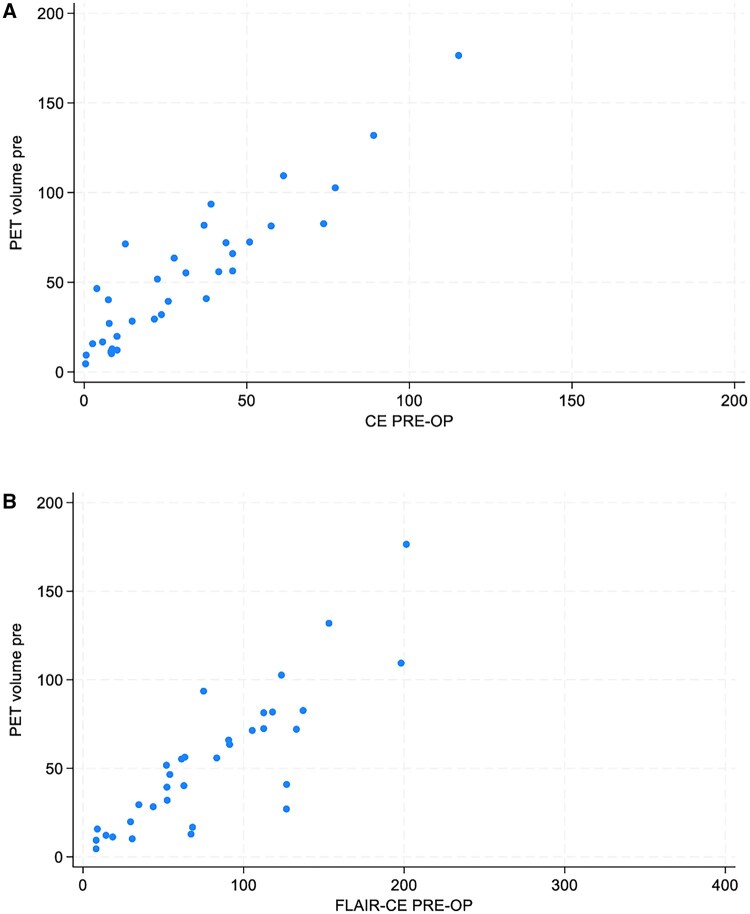
Correlation between preoperative volumes and PET volume. (A) Variables: preoperative contrast enhancement tumor volume (CE) and preoperative PET volume (Pearson’s *r*  =  0.9). (B) Variables: preoperative PET and preoperative FLAIR-TV (Pearson’s *r*  =  0.8).

Statistical analyses showed a significant correlation between preoperative FLAIR-TV and preoperative 11[C]-MET PET volumes (Pearson’s *r*  =  0.9). We also found a significant correlation between preoperative CE volumes and preoperative ^11^C-MET PET volumes (Pearson’s *r*  =  0.84) (Figure 1).

Regarding preoperative volumes, no significant difference was found between the CR + NTR cluster and the SUPR group for CE-volume and FLAIR-TV (*P  *=  .26, .12, and .13).

### Survival Analysis

We conducted a survival analysis to compare the outcomes of patients who underwent PET-guided resection with those who underwent MRI-guided resection according to the new RANO criteria classification system, operated on at our institute. There were no significant differences between the MRI and PET groups in terms of sex, age, MGMT methylation status, tumor location, tumor side, or preoperative KPS. However, the PET group had significantly larger preoperative FLAIR volumes (*P*  <  .001). The median PFS was 14.94 months for PET, with a significant difference with SUPR (*P*  =  .0003). Median OS was 21.88 months, with a significant difference with the SUPR group (*P*  =  .0254).

Median OS was 15, 17, and 24 months for NTR, CR, and SUPR, respectively, with a significant difference between NTR and SUPR group (HR 0.471, 95%CI 0.301 to 0.735, *P  *=  .001), and no difference between CR and NTR (HR 0.882, 95%CI 0.554 to 1.402, *P  *=  .595). There was no significant difference between CR, NTR, and PET groups for both PFS and OS (respectively, *P*  =  .191 and *P*  =  .205).

**Figure 2.**
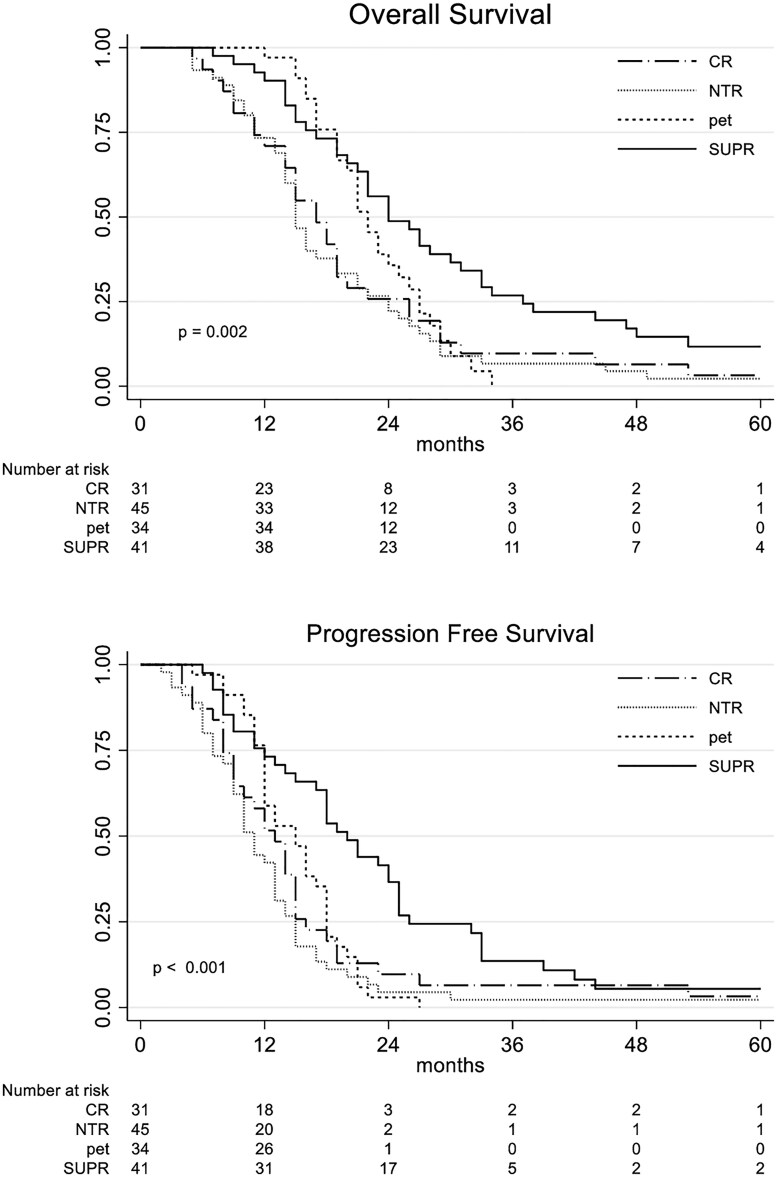
Overall and progression free survival in the PET group and the MRI-guided groups.

The results of the univariate Kaplan–Meier model analysis are shown in Figure 2.

## Discussion

Maximizing EOR remains one of the most effective strategies for managing IDH-wildtype GBM. The RANO group’s concept of supramaximal resection (SUPR) promotes removing both contrast-enhancing and FLAIR hyperintense regions to more effectively address infiltrative tumor margins.

Based on this background, we investigated whether the use of ^11^C-MET PET and MRI could provide additional insights that impact surgical decision-making.

In our prospective study, we enrolled 34 patients (22M, 12F, M: F ratio 1.83:1) with IDH1-wildtype GBM who underwent a complete PET-guided resection. Our volumetric analyses support previous findings that MET-PET surpasses MRI in detecting infiltrative tumor margins.^6^^,^^8^^,^^9^^,^^14^ The spatial overlap between MET-PET and FLAIR volumes (91.17% of cases) underscores MET-PET’s ability to map edema-associated infiltration, consistent with studies showing MET-PET’s superiority over FLAIR for identifying non-enhancing glioma regions. However, the 8.82% of cases where MET-PET volumes exceeded FLAIR highlights metabolic activity in areas without T2/FLAIR hyperintensity phenomenon observed in gliomas with aggressive subclones. The strong MET-PET/contrast-enhancement correlation (*r* = 0.84) further supports its role in targeting active tumor cores, as methionine uptake correlates with proliferative area. This finding reinforces the observation that metabolic activation often exceeds the areas of contrast enhancement, revealing tumor infiltration in regions deemed non-contrast enhancement on MRI.^4^^,^^7^^,^^15^

The objective of our study was to assess the impact of metabolic imaging-guided surgical resection versus qualitative MRI-based assessments, particularly comparing outcomes between PET-guided resection and SUPR according to the new RANO criteria^2^ at our institute. No significant differences were observed between the SUPR and PET groups regarding sex, age, MGMT methylation status, tumor location, tumor side, or preoperative Karnofsky Performance Status (KPS). Both groups had a similar distribution of lesions in eloquent regions. However, the PET group had larger preoperative FLAIR volumes (*P*  <  .001) and a higher proportion of highly diffuse tumors, while the SUPR group had more nodular tumors. Interestingly, the SUPR group demonstrated a statistically significant difference in both OS (*P*  =  .0254) and PFS (*P*  =  .0003) compared to the PET group, while the PET group showed improved OS ad PFS, although not statistically significant, compared to the CR groupA potential explanation could be related to the behavior of glioma stem cells (GSCs), which play a well-known critical role in resistance to conventional therapies, including surgery and chemotherapy.^16^^,^^17^ One key factor contributing to their resilience is their unique metabolic profile. Unlike differentiated glioma cells, GSCs are highly adaptable and can switch between various metabolic states depending on their environment.^18–21^ This adaptability enables them to persist in less favorable conditions, such as low oxygen or nutrient levels, commonly found in tumors. One hypothesis suggests that GSCs may be less metabolically active and therefore less detectable by PET imaging, which relies on radiolabeled molecules to target metabolically active cells. GSCs often depend more on oxidative phosphorylation and other alternative pathways rather than the glycolytic metabolism observed in rapidly proliferating tumor cells. This metabolic quiescence can make them less visible to imaging techniques like PET, which primarily detect metabolically active cells. In contrast, SUPR, which involves resecting tissue beyond the visible tumor boundary, may include areas with these metabolically quiescent GSCs, potentially leading to improved survival outcomes (both OS and PFS) by removing more of these resistant cells. Thus, while PET-guided surgery is precise in targeting active tumor regions, it may overlook these metabolically silent but clinically significant GSC populations.

Our data support a nuanced interpretation: while PET imaging remains a valuable tool for defining metabolically active tumors, it may not fully capture the infiltrative and heterogeneous nature of GBM. As such, its role should be viewed as complementary rather than exclusive. The incorporation of artificial intelligence and machine learning could further enhance the power of MET-PET and FLAIR MRI.

Integrating MET-PET with advanced MRI techniques (e.g., diffusion tensor imaging) and GSC-specific biomarkers could enhance resection accuracy. Randomized trials comparing PET-guided surgery to SUPR are warranted, particularly using hybrid PET/MRI systems for real-time metabolic navigation. Additionally, exploring dynamic MET-PET parameters (e.g., time-to-peak) may improve the detection of metabolically heterogeneous subregions. This could pave the way for personalized treatment strategies tailored to the individual patient’s tumor characteristics.

### Limitation

This study has several limitations that need to be acknowledged. First, the lack of randomization limits the overall quality of the evidence, introducing potential selection and clinical bias. While randomized trials could provide more robust insights for clinical decision-making, they were not feasible in this case due to ethical and practical constraints. Additionally, the study is limited by its small sample size, which may affect the ability to generalize the results. To address these intrinsic limitations, we focused on minimizing variables associated with tumor characteristics and treatment methods. We decided to limit our analysis to newly diagnosed IDH1 wildtype GBM patients to emphasize cohort homogeneity. It is important to note that surgical treatments were performed within a relatively short timeframe, systematically employing intraoperative neurophysiological mapping when necessary. There is also the possibility of human error in radiographic measurements. Even with careful methodology and validation by 2 independent reviewers, our volumetric measurements are still subject to limitations such as slice thickness and the computational restrictions of the software used. Finally, ^11^C-MET PET is available in only a few specialized neuro-oncology centers because the short half-life of ^11^C (20 minutes) necessitates the presence of an onsite cyclotron. In Europe, ^11^C-MET has largely been replaced by [18F]-FET and [18F]-FDOPA. However, as MET, FET, and FDOPA are all transported by the same L-amino acid transport system, similar diagnostic results can generally be expected among these radiotracers.

## Conclusions

Our findings suggest that SUPR, guided by anatomical imaging including FLAIR MRI, offers superior survival benefits compared to PET-guided approaches alone. The study provides evidence that, while ^11^C-MET PET offers superior sensitivity in detecting metabolically active tumor regions beyond the contrast-enhancing core, it may not fully capture the infiltrative margins that harbor treatment-resistant subpopulations, such as GCSs. The role of PET should be viewed as complementary rather than exclusive in surgical planning. Future studies should focus on integrating PET with advanced MRI modalities and molecular markers to enhance tumor delineation.

## Data Availability

The datasets generated during and/or analyzed during the current study are available from the corresponding author on reasonable request.
